# Retraction and republication: The roles of a Grandmother in African societies – please do not send them to old people’s homes

**DOI:** 10.7189/jogh.10.010105

**Published:** 2020-06

**Authors:** The Editors of the Journal of Global Health

In the June 2019 issue of the *Journal of Global Health* published a viewpoint “The roles of a Grandmother in African societies – please do not send them to old people’s homes” [1]. After its publication, concerns were raised about the possible text similarity. A mutual agreement between the complainant, authors and the editors was reached to retract the viewpoint and publish the corrected version [2]. The corrections do not change the message of the viewpoint. In the **Online Supplementary Document** we include the letter from the authors, and the original article with authors’ annotations.

## Additional material


Online Supplementary Document
