# Supplementary material for: Retraction and republication: The roles of a Grandmother in African societies – please do not send them to old people’s homes
Source: J Glob Health. 2020 Jun 17;10(1):010105. doi: 10.7189/jogh.10.010105 (PMC7323933; doi:10.7189/jogh.10.010105)
Supplement: Online Supplementary Document [file jogh-10-010105-s001.pdf]

Swiss TPH

6 December 2019

The Editor JOGH

Dear Editor

<u>RE: RETRACTION AND REPUBLICATION OF AN AMENDED VIEWPOINT</u>

We wish to retract the view point, "The roles of a Grandmother in African societies – please do not send them to old people's homes," to republish an amended viewpoint.

Janet Michel, Astrid Stuckelberger, Fabrizio Tediosi, David Evans, Piet van Eeuwijk

JOGH 2019; 9: 010306.

***Editorial statement on the retraction;**

To the Editor

We would like to retract the view point, "The roles of a Grandmother in African societies – please do not send them to old people's homes," and to republish an amended viewpoint.

Janet Michel, Astrid Stuckelberger, Fabrizio Tediosi, David Evans, Piet van Eeuwijk

JOGH 2019; 9: 010306.

***A statement of what was wrong with the previous article;**

The reasons for retraction are as follows;

- *<u>Source 4 is a newspaper, and the original paper about which the news article was written has been retracted for plagiarism</u>* (https://www.ncbi.nlm.nih.gov/pmc/articles/PMC5757466/). Plagiarism in "What is the role of a Grandmother in a Malawian society and how can we as health care workers support her?' (MMJ 2007;19[3]:126–127)

We were not aware of this and we now have replaced this reference with a new one including the original source Aubel J.

[5] Old age in Africa - Research Seminar. Afr Stud Cent Leiden n.d. https://www.ascleiden.nl/news/old-age-africa-research-seminar (accessed December 4, 2019).

[18] Aubel J. The role and influence of grandmothers on child nutrition: culturally designated advisors and caregivers. Matern Child Nutr 2012; 8:19–35. https://doi.org/10.1111/j.1740-8709.2011.00333.x.

- *<u>Reference 3 Marg News. The annual newsletter of the Medical Anthropology Research Group University of Basel. FROM CURE TO CARE Project Leader van Eeuwijk.</u>* Available: https://www.swisstph.ch/fileadmin/user_upload/SwissTPH/Institute/EPH/

Society_Gender_and_Health/MARG_News_2014.pdf. Accessed: 3 October 2018.

removed as it could not be found.

A new reference has been added

[5] Old age in Africa - Research Seminar. Afr Stud Cent Leiden n.d. https://www.ascleiden.nl/news/old-age-africa-research-seminar (accessed December 4, 2019).

- *Source 2 WHO reports as references;*

This paragraph was not paraphrased and The World Health report Ageing and Health was not correctly referenced and has now been included in the reference list alongside WHO ageing and life course report as well as WHO | Ageing and Life Course. WHO n.d. http://www.who.int/ageing/en/ (accessed December 4, 2019).

- *Source 3 Illinois University. Characteristics of a Great Babysitter. 2017.* From:https://extension.illinois.edu/babysitting/characteristics. cfm. Accessed 3 October 2018.

This reference we used could not be found. This has now been replaced with a new reference

[20] What to Look For in a Babysitter (12 Essential Qualities). Kidsit Babysitt Tips n.d. https://kidsit.com/essential-babysitter-qualities-to-look-for (accessed December 4, 2019).

- *Source of an article from other authors. There is a citation, but without paraphrasing.*

Paraphrasing has been done and other references added and now reads; Older people continue to have aspirations to well-being and respect regardless of declines in physical and mental capacity [2,9,10].

[2] WHO | What is Healthy Ageing? WHO n.d. http://www.who.int/ageing/healthy-ageing/en/ (accessed December 4, 2019).

[9] WHO | Ageing and Life Course. WHO n.d. http://www.who.int/ageing/en/ (accessed December 4, 2019).

[10] Astrid Stuckelberger PD. Transgenerational Perspective on Peace and on Violence Prevention Role of Older Persons and Grandparents in the Culture and Development of Peace and Non-Violence n.d. (accessed December 4, 2019)

***The retracted article, with the changes highlighted.**

The retracted article with changes highlighted is attached.

We apologize for any inconveniences caused.

Yours sincerely

Janet Michel

© 2019 The Author(s)
JoGH © 2019 EUGHS

VIEWPOINTS

The roles of a Grandmother in African societies – please do not send them to old people's homes

Janet Michel[1], Astrid Stuckelberger[2], Fabrizio Tediosi[1], David Evans[1], Piet van Eeuwijk[3]

[1] Health Systems and Policy Department University of Basel, Basel, Switzerland
[2] Institute of Global Health, University of Geneva, Geneva, Switzerland
[3] Medical Anthropology Department, University of Basel, Basel, Switzerland

October 1 is the International Day of Older Persons, a recognition that has been in place for over 20 years now, but I must say very few stop to commemorate this day –myself included.

The day is supposed to be celebrated by raising awareness about issues affecting the elderly and to appreciate the contributions that older people make to society [1]. What is the one thing that most of us today are going to become? – get older [2]. Populations around the world are rapidly ageing. Ageing presents both challenges and opportunities. Societies that adapt to this changing demographic and invest in *Healthy Ageing* can enable individuals to live both longer and healthier lives and for societies to reap the dividends. 80% of people over 60 will live in low- and middle-income countries by 2050. In Africa, old age is a social category experienced in relation to other generations, especially to youth [3] while as in Europe old peoples' homes have been established and more or less accepted as the living arrangement for the elderly. This is however without draw backs. Though moving into a nursing home is an individual experience [4], people who move into a nursing home experience different types of changes which they feel to a greater or lesser degree as stressful. The change in social status, the impact on autonomy, the feeling of having no place to call home, the change in social contacts, and the reduction of habitual activities rank first in the presentation of the results and endanger the people's identity which they had before [4,5]. Nursing home residents have experiences which they perceive as compulsive and degrading [6].

It starts with us-envisage yourself as an elderly. Where would you rather be? Love and a sense of belonging matter to all humans, and seem to matter to the end. There seems to be no cut off point. We owe our grandmothers so much, we can never repay them. The least that Africa can do to say thank you, is to keep them at home as far as possible. We can only reiterate the following: "Family is the most important thing in the world" (Princess Diana).

VIEWPOINTS

Nursing home residents still want to feel part of society and also wish to remain in contact with family members [5]. Sadly, nursing home residents hardly make use of their own capabilities because they feel that their abilities will be insufficiently recognised [5]. Healthy Ageing is about creating the environments and opportunities that enable people to be and do what they value throughout their lives. Everybody can experience Healthy Ageing. Being free of disease or infirmity is not a requirement for Healthy Ageing as many older adults have one or more health conditions, that when well controlled, have little influence on their well-being. Health and well-being are determined not only by our genes and personal characteristics but also by the physical and social environments in which we live. Environments play an important role in determining our physical and mental capacity across a person's life course and into older age and also how well we adjust to loss of function and other forms of adversity that we may experience at different stages of life, in particular in later years [2,7]. Ageing is not a problem to be fixed nor a disease to be treated but a natural and powerful life process [2].

The 'longer life phenomenon' comes forward with major improvements in the ageing process of the individual. People all around the world are getting older, are in better health and remain active longer, thus play a longer lasting role in the family and can contribute longer to the social system [8].

Traditionally in Africa, families are the primary care provider for the elder generation thereby promoting societal solidarity among its generations. Research has revealed that families are willing to provide care for the elder generation [3] and this should be capitalized on. Africa does not have to repeat the mistakes made in the west of institutionalizing the elderly [5,6]. Already today many older people complain that they do not feel respected by today's youth and they feel their authoritative position being undermined as they are progressively no longer considered as responsible for upbringing, educating and disciplining the younger generation [3].

Ageism is the stereotyping, prejudice, and discrimination against people on the basis of their age. Ageism is widespread and an insidious practice which has harmful effects on the health of older adults [2]. For older people, ageism is an everyday challenge. Overlooked for employment, restricted from social services and stereotyped in the media, ageism marginalises and excludes older people in their communities. Ageism is everywhere, yet it is the most socially normalized form of prejudice, and is not widely countered – like racism or sexism. These attitudes lead to the marginalisation of older people within our communities and have negative impacts on their health and well-being [7].

> We view this as systems thinking in a society. A dysfunction in one part e.g. the elderly will inevitably lead to the dysfunction of the whole system – society. Ambuya, Mbuya, Bibi, Gogo – we call them by different names in Southern Africa, but we are unified by what these women mean to us, our families and societies.
>
> This resource should be capitalized on rather than locked away in old people's homes. In the same token we celebrate Father's day and Mother's day – we urgently need to consider and acknowledge these Grandmothers.
>
> Let's all unite behind a call for a Grandmothers day, a holiday in Africa may be the world, where all grandkids can travel to their grandparents and thank them for all their love and sacrifices.

We cannot challenge bias unless we are aware of it [2]. It is therefore is important to acknowledge the fundamental roles older persons in Africa play in the society and arrest this growing phenomenon-Ageism [1]. In spite of bemoaning the loss of strength, older people praise the benefits and blessings of old age. They are proud of taking up responsibilities, engagements, and duties, economically as well as socially, albeit in a different way than in the past [3]. It is a sense of purpose that gets eroded first when they get institutionalised. One example-the role of grand-parenthood and of older persons is too often not considered or underestimated when addressing the younger generations' problems, or when building a sustainable family and social policy. Longevity is a fundamental hallmark of human progress [2], so we ought to celebrate it. Below we explore and describe the roles Grandmothers in Africa play (**Figure 1**), which we should acknowledge, highlight and celebrate.

HERBALIST

Grandmothers share a body of knowledge and skills concerning illness and herbal treatments. This is learned as part of growing up in a rural home where mothers and grandmothers use herbal medicines in dealing with illness. It is learned through relationship with grandmother. Healing is embedded in the close relationship of reciprocity and care between grandmothers, mother and grandchildren. Through shared daily life with grandmother, mother, grandchild develops social sense, respect, and compassion for people, as

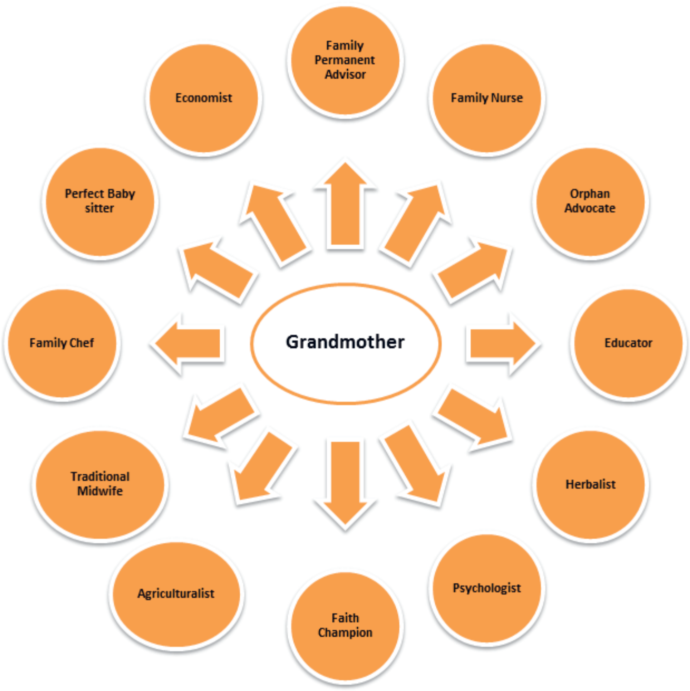

Figure 1. Summary of roles played by grandmothers in Africa.

well as practical skills to heal. Learning to heal is not only embedded in everyday practice and in social relations, but is also a moral and emotional process in Africa [9].

THE PSYCHOLOGIST: COMFORTER AT HOME AND FUNERALS

Funerals in Africa are not only an occasion to mourn. They are also an opportunity to celebrate the life of the dearly departed. Funerals are a social event attended by a large number of mourners, which could reach hundreds–the more, the better [10].

For a funeral to proceed as a celebration, something needs to have happened-that means turning that loss into a gain, turning the life lost into gratitude that the person lived in the first place. Doing the background jobs, are usually the elderly women of the village who take up the role of the "Sahwira" meaning making light of the situation and bringing glimpses of joy in a situation that is supposed to be dark. The Sahwiras put on the clothes of the deceased, play the jokes the deceased played, imitate him/her doing things like walk like him or her, prompting the mourners to take a break and cherish the time they spent with the deceased.

Putting the elderly into the old peoples' home would deprive the community of such free psychological treatment to the family and community. Research has revealed that absent or dysfunctional "grand-parents" model in a family could have effects on the psychological development of children, similar to becoming "grand-orphans" and thus lacking the possibility to integrate core values of life/death in their own life development, which could lead to disruptive behaviour [8].

THE FAITH CHAMPION

Grandparents tend to be the encouragers, the faith power houses grandchildren and everyone turns to when life throws itself at us. With their life experience, they have wisdom to calm us and assure us that life happens but do not ever give up, there is sunshine after rain [11]. Mothers' unions from different denominations are powerhouses to encourage young women, widows and orphans to soldier on despite their current circumstances. The praying together, the singing together, the crying together, the celebrating together and the mourning together, fosters a strong intergenerational bond among women that would not be possible if the elderly would be bundled into old people's or nursing homes.

Children and grandchildren faced by life storms often share them with grandparents and the assurance grandmothers give eg, by saying, "Let's pray and I will continue to pray for you" has an indescribable effect on how one carries on despite the rain. When they meet during mothers union gatherings, one of the tasks on their to-do list is to pray for their children and grandchildren-making them role models as well as prayer champions. It is their love, devotion and determination that make the difference in our lives [12].

THE AGRICULTURALIST

What to plant, when to plant and how to plant is critical for a harvest in Africa where the effects of global warming are being felt. Grandmothers know when to plant beans and how to plant sweet potatoes and groundnuts that need covering when they start to flower. They know which crops need little rain and which ones need to be planted in swamps. All this knowledge can only be passed on while living and working beside a grandmother. These grandmothers know how to produce process and conserve food eg, dried vegetables. They have kept the knowledge and skills needed to save and breed indigenous seeds [13]. Taking them away means losing such invaluable knowledge.

THE FAMILY EDUCATOR

Ancient cultures recognized the older generations as the source of knowledge and wisdom and referred to them as models for their own lives and future. The Elders were praised as "Transmitters of culture", as "Guardians of the secrets of life" or as "the Wise" to consult in the prevention of conflicts and preservation of peace in the individual, family and in society [8]. When it comes to cultural values they know them best. They know why a man has to pay lobola and why one has to wait with sex till marriage. Believe me, they have seen it all and the current divorce rates could give us a hint. Some values might seem ridiculous at face value but its only through going deeper that one starts to appreciate it all. Why should sexual relationships be restricted- a look at the HIV/AIDS figures will make one appreciate the grandmothers' wisdom, if only both parties were to take heed. Seemingly small things, like simply encouraging the younger generation to be disciplined and to work hard is a role these grandmothers play daily [11]. They also have a role in maintenance and nurturing grandchildren to grow up valuing traditional morals and beliefs [14]. Grandmothers are a source of inspiration for many in the family. They are therefore in a position to set standards and goals for grandchildren, particularly if they themselves have been high achievers in their life [14].

The socio-economic interdependency of generations in building a sustainable society calls for a systems approach where we value interdependency [8]. Wisdom has been considered one of the highest forms of knowledge and personal functioning all throughout the history of mankind [8]. Older people are key to violence prevention and to the promotion of a culture of peace. The increase in violence in schools and in youth in general concerns the whole of society as violence in youth is but an expression of a dysfunctional society, a symptom of an unbalance that older generations have a duty and opportunity to contribute to [8].

THE ECONOMIST

African women have always contributed to the economy of the home [15].Who-ever would like to get some lessons on saving and self -sufficiency should spend some time with a grandmother in her rural home. All that is served is produced by her, the cooking oil, dovi (peanut butter), the mealie meal-all produced by her. You can spend weeks there and never have to reach for your wallet. Milk is produced by her cows and bread is home-made and meat from her goats, chickens and even cows. Big saving and self-sufficiency lessons can be learnt from these grandmothers.

THE PERFECT BABY SITTER

The importance of grandparents in raising grandchildren is not trivial and gaining importance as they provide grandchildren care- from babysitting to being a custodial grandparent [8]. The qualities of a perfect baby sitter include being dependable, responsible, love little children, be self-confident, mature,

knowledgeable and safety-conscious [[illegible] the list describes the grandmothers very well. In traditional cultures, grandparents often have a direct and clear role in relation to the care and nurture of children [14]. The grandmothers I know fulfil and exceed all the above mentioned qualities and at no additional cost for that matter, so why send them to old people's homes?

THE FAMILY CHEF

Grandmothers' food is the best; all grandkids know that and cannot wait until holidays arrive. The kitchen knowledge and experience grandmothers have is valuable and should not be taken for granted. In African societies, traditional recipes will be and are quickly getting lost as parents live in cities far away from grandparents. Putting the elderly in old peoples' homes would further erode this knowledge.

They know best how to prepare wild vegetables now known as biological vegetables, how to prepare insects, (ishwa) now known as superfoods with super protein [17]. The grandmothers make this freely available to us and we ought to preserve such a heritage. Grandmothers play a central role as advisers to younger women and as caregivers of both women and children on nutrition and health issues influencing maternal and child practices, specifically regarding pregnancy, feeding and care of infants, young children and the sick [18]. This knowledge is priceless.

THE FAMILY NURSE

Grandmothers are 24 hours available for the family. When a family member falls ill, they avail themselves, at no cost and provide 24hrs care. Their wisdom is calming, their faith gives one hope and they know the type of food and how to prepare it to whet appetites. We need the grandmothers in the family. They have played great nursing roles during the HIV/AIDS pandemic [14] and we need them for more challenges to come.

THE MIDWIFE

Throughout history, traditional birth attendants have been the main health care providers for women during childbirth in Africa. They attend to the majority of deliveries in the rural areas of developing countries. When a woman is pregnant, who best can answer all the questions except grand mum, who herself has undergone the experience if not several times and over the years has accompanied many. Traditional midwives have been an integral part of African medicine for centuries. Because African people still love and fear spirits, they bring in a complementary component to conventional health care service, hence they are highly respected in African communities as they perform cultural rituals and provide essential social support to women during childbirth [19,20]. With additional formal training, traditional midwives, these grandmothers can be considered an important part of the informal community health care system [19].

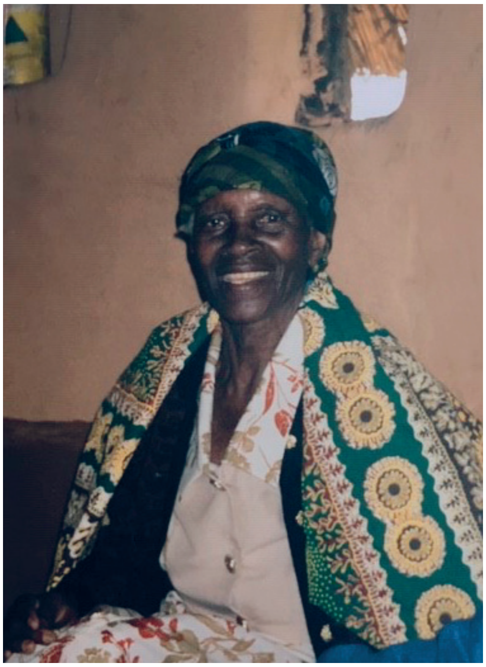

Photo: by J Michel in 2003 (used with permission). Smiling, valued and useful to the end – despite Parkinsonism and mobility issues. My wish for every-one getting older (meaning all of us).

THE ORPHAN ADVOCATE

Grandparents—grandmothers in particular, are Africa's great hope. In Africa, grandmothers cannot be taken for granted because they are essential to the survival of 13 million children orphaned in the AIDS pandemic. Orphanages are not part of African culture; orphans look to family members to take them in, and since many of their parents' generation have died of AIDS, it is grandmothers who look after 40 to 60 percent of them. Without grandparents, children are often left to fend for themselves [14,21].

This resource should be capitalized on rather than locked away in old people's homes. In the same token we celebrate Father's day and Mother's day- we urgently need to consider and acknowledge these Grandmothers.

A Grandmothers day, a holiday in Africa may be the world, where all grandkids can travel to their grandparents and thank them for all their love and sacrifices is long overdue in our view.

PERMANENT FAMILY ADVISOR AND CHEER LEADER

The relationship to grandparents is different to that of parent-child because it is often uncluttered by guilt, resentment or confused loyalties - all the problems that can arise in a straight parental relationship. Grandmothers have a prominent and influential role within the family [14].

They have seen it all and they know that nothing is permanent. They know how to comfort, they know when to and how to confront ad and they also know when and how to counsel. Invaluable and impartial advice to each and every family member when-ever needed. Our Grandmothers are indeed a global phenomenon and we should celebrate them and keep them in the family [22].

CONCLUSIONS

Loneliness and feeling of valueless in old people's homes have been widely documented [5,6]. Older people continue to have aspirations to well-being and respect regardless of declines in physical and mental capacity [3,7]. Families in Africa where 80% of the elderly will be resident in 2050 still view and accept that the elderly have a role to play in the family and society. Highlighting these invaluable roles they contribute to society, is a first step in fighting the rising ageism and abuse of elderly [7]. There are reciprocal and enriching benefits in keeping the elderly at home. Ageism is a global human rights issue [2] the longer we wait, the more damage it does to ourselves and our place in the world [2].

Acknowledgements: We would like to acknowledge Prof. Dr Brigit Obrist for reading the first draft and for giving me confidence and encouragement to write and publish.

Funding: None.

Authorship contributions: JM contributed to the initial concept of paper and wrote the first draft. AS, FT, DE and PE commented and contributed to subsequent drafts.

Competing interests: The authors have completed the Unified Competing Interest form at www.icmje.org/coi_disclosure.pdf (available on request from the corresponding author) and declare no conflict of interest.

REFERENCES

1 REPSSI. Recognising the role of older persons in African society. Available: http://www.socialserviceworkforce.org/system/files/resource/files/Psychosocial%20care%20and%20support%20for%20older%20carers%20of%20orphaned%20and%20vulnerable%20children%20Policy%20guidelines.pdf. Accessed: 3 October 2018.

2 Applewhite A. Let's end ageism. Tedx. 2017. Available: https://www.youtube.com/watch?v=WfjzkO6_DEI&t=14s. Accessed: 3 October 2018.

3 Marg News. The annual newsletter of the Medical Anthropology Research Group University of Basel. FROM CURE TO CARE Project Leader van Eeuwijk. Available: https://www.swisstph.ch/fileadmin/user_upload/SwissTPH/Institute/EPH/Society_Gender_and_Health/MARG_News_2014.pdf. Accessed: 3 October 2018.

4 Porter EJ, Clinton JF. Adjusting to the nursing home. West J Nurs Res. 1992;14:464-77. Medline:1509732 doi:10.1177/019394599201400404

5 Riedl M, Mantovan F, Them C. Being a nursing home resident: A challenge to one's identity. Nurs Res Pract. 2013;2013:932381. Medline:23691302

6 Fiveash B. The experience of nursing home life. Int J Nurs Pract. 1998;4:166-74. Medline:9844091 doi:10.1046/j.1440-172X.1998.00062.x

7 World Health Organization. Ageing and life course. Geneva: World Health Organisation: 2018.

8 Stuckelberger A. A transgenerational perspective on peace and on violence prevention role of older persons and grand-parents in the culture and development of peace and non-violence. New York Academy of Science 2005: Kluwer Edition.

9 Prince R, Geissler PW. Becoming "one who treats": a case study of a luo healer and her grandson in Western Kenya. Anthropol Educ Q. 2001;32:447-71. doi:10.1525/aeq.2001.32.4.447

10 Newton P. The long goodbye: Why funerals are big deals in Ghana. CNN 2014.Available: http://edition.cnn.com/2014/03/11/world/africa/on-the-road-ghana-funerals/index.html. Accessed 3 October 2018.

11 Wayde van Niekerk CNN. The 74-year-old coach behind South African's golden run at Rio 2016, 2016. Available: https://edition.cnn.com/2016/08/15/sport/news-rio-olympics-van-niekerk-grandma-coach/index.html. Accessed: 3 October 2018.

12 Norwood D. Praying Grandmothers. 2004. Available: https://www.youtube.com/watch?v=_a57TFkxNy8. Accessed: 3 October 2018.

13 Kim HJ. FOOD SOVEREIGNTY: TAKING ROOT IN WOMEN'S KNOWLEDGE. 2017. Available: http://edepot.wur.nl/416219. Accessed 3 October 2018.

14 Aubel J. The role and influence of grandmothers on child nutrition: culturally designated advisors and caregivers. Matern Child Nutr. 2012;8:19-35? Matern Child Nutr. 2012;8:19-35. Medline:21951995 doi:10.1111/j.1740-8709.2011.00333.x

15 Magadla S. Matrifocality and shared motherhood, in Mail and Guardian 2017. Available: https://mg.co.za/article/2017-08-25-00-matrifocality-and-shared-motherhood. Accessed: 3 October 2018.

16 Illinois University. Characteristics of a Great Babysitt[illegible]017. Available: https://m.extension.illinois.edu/babysitting/characteristics.cfm. Accessed 3 October 2018.

17 van Huis A, Oonincx DGAB. The environmental sustainability of insects as food and feed. A review. Agron Sustain Dev. 2017;37:43. doi:10.1007/s13593-017-0452-8

18 Aubel J. The role and influence of grandmothers on child nutrition: culturally designated advisors and caregivers. Matern Child Nutr. 2012;8:19-35. Medline:21951995 doi:10.1111/j.1740-8709.2011.00333.x

19 AMREF. Amref Health Africa's Position on the Role and Services of Traditional Birth Attendants. 2017. Available: https://amref.org/news/amref-health-africas-position-statement-on-traditional-birth-attendants. Accessed: 3 October 2018.

20 Troskie TR. The importance of traditional midwives in the delivery of health care in the Republic of South Africa. Curationis. 1997;20:15-20. Medline:9287548

21 Lewis Kulzer J, Penner JA, Marima R, Oyaro P, Oyanga AO, Shade SB, et al. A group of grandparents reaches out to help alleviate an HIV/AIDS epidemic. J Int AIDS Soc. 2012;15:8. Medline:22353553 doi:10.1186/1758-2652-15-8

22 Gianturco P. Grandmother Power: A Global Phenomenon. Brooklyn NY: Powerhouse Books; 2012.

Correspondence to:
Janet Michel
Bahnhofstrasse 75
Roggwil 4914
Switzerland
janetmichel71@gmail.com
